# Health Tract, No. 16

**Published:** 1862-01

**Authors:** 


### MEASLES AND CONSUMPTION.

(*From Hall's Journal of Health*, 42 *Irving Place*, *New-York*.)

THIS disease prevails extensively in cities during the winter season, and will usually cure itself, if only protected against adverse influences. The older persons are, the less likely they are to recover perfectly from this ailment, for it very often leaves some life-long malady behind it. The most hopeless forms of consumptive disease are often the result of ill-conducted or badly managed measles. In nine cases out of ten, not a particle of any medicine is needed.

Our first advice is, always, and under all circumstances, send at once for an experienced physician. Meanwhile keep the patient in a cool, dry, and well-aired room, with moderate covering, in a position where there will be no exposure to drafts of air. The thermometer should range at about sixty-five degrees, where the bed stands, which should be moderately hard, of shucks, straw, or curled hair. Gratify the instinct for cold water and lemonade. It is safest to keep the bed for several days after the rash has begun to die away. The diet should be light, and of an opening, cooling character.

The main object of this article is to warn persons that the greater danger is after the disappearance of the measles. We would advise that for three weeks after the patient is well enough to leave his bed, he should not go out of the house, nor stand or sit for a single minute near an open window or door, nor wash any part of the person in cold water nor warm, but to wipe the face with a damp cloth. For a good part of this time the appetite should not be wholly gratified; the patient should eat slowly of light nutritious food. In one case, a little child, almost entirely well of the measles, got to playing with its hands in cold water; it gradually dwindled away and died. All exercise should be moderate, in order to prevent cooling off too quickly afterwards, and to save the danger of exposure to drafts of air, which, by chilling the surface, causes *chronic diarrhœa*, if it falls on the bowels; *deafness for life*, if it falls on the ear; or *incurable consumption*, if it falls on the lungs.

---

THE easiest method of securing an erect and manly carriage is to walk with the chin slightly above a horizontal line, as if looking at something higher than your own head.

